# Genome Sequence of *Serratia plymuthica* RVH1, Isolated from a Raw Vegetable-Processing Line

**DOI:** 10.1128/genomeA.00021-14

**Published:** 2014-02-06

**Authors:** Rob Van Houdt, Daniel Van der Lelie, Javier A. Izquierdo, Abram Aertsen, Joleen Masschelein, Rob Lavigne, Chris W. Michiels, Safiyh Taghavi

**Affiliations:** a Unit of Microbiology, Belgian Nuclear Research Centre (SCK∙CEN), Boeretang, Mol, Belgium; b Center for Agricultural and Environmental Biotechnology, RTI International, Research Triangle Park, North Carolina, USA; c Laboratory of Food Microbiology, KU Leuven, Leuven, Belgium; d Laboratory of Gene Technology, KU Leuven, Leuven, Belgium

## Abstract

We announce the genome sequence of *Serratia plymuthica* strain RVH1, a psychroloterant strain that was isolated from a raw vegetable-processing line and that regulates the production of primary metabolites (acetoin and butanediol), antibiotics, and extracellular enzymes through quorum sensing.

## GENOME ANNOUNCEMENT

*Serratia plymuthica* RVH1 is a Gram-negative bacterium (*Enterobacteriaceae* family) isolated from a raw vegetable-processing line in an industrial kitchen (1, 2). Prevalent in soil, air, and water, *Serratia* species are commonly associated with raw food materials and are often implicated in the spoilage of various foods. In addition, as opportunistic pathogens, they pose a food-borne health hazard and cause nosocomial infections. *S. plymuthica* RVH1 is being studied for its psychrotolerance mechanisms and its LuxR-LuxI-type quorum-sensing (QS) system based on *N*-acyl-l-homoserine lactones (3). The latter controls the production of primary metabolites (switch from mixed acid to acetoin and butanediol fermentation) (4, 5), the polyamino antibiotics zeamine, zeamine I, and zeamine II (6), and extracellular enzymes (7, 8). These QS-regulated traits provide a selective advantage in challenging and competitive environments (5, 7, 9).

Full-genome sequencing was performed using Illumina data (10). Illumina short-insert (average insert size, 270 bp) and long-insert (average insert size ± standard deviation, 9,850 ± 2,377 bp) paired-end libraries generated 18,135,900 and 69,799,586 reads, respectively, totaling 9.7 Mbp of Illumina data (1,759× coverage). The initial draft data (21 contigs in 5 scaffolds) was assembled with AllPaths (version 39750) and Velvet (version 1.1.05 [11]), and the consensus was computationally shredded into 10-kbp and 1.5-kbp overlapping shreds, respectively. The Illumina data were reassembled with Velvet using the first Velvet assembly to guide the next assembly and subsequently shredded into 1.5-kbp overlapping fake reads. Fake reads from the AllPaths assembly and both Velvet assemblies, as well as a subset of the Illumina CLIP paired-end reads, were assembled using parallel Phrap, version 4.24 (High Performance Software, LLC). Possible misassemblies were corrected with manual editing in Consed (12–14). Gap closure was accomplished using repeat resolution software (W. Gu, unpublished data) and sequencing of bridging PCR fragments with Sanger and/or PacBio (C. Han, unpublished data) technologies. A total of 40 additional sequencing reactions, five PCR PacBio consensus sequences, and no-shatter libraries were completed to close gaps and to raise the quality of the final sequence. Within the sequence, a comparison to a sequenced bacterial artificial chromosome (BAC) clone region spanning 170 kb, associated with the identification of the zeamine antibiotics (8), revealed no sequencing inconsistencies.

The genome has a 5,514,320-bp-long chromosome, with a G+C content of 56.2%. Of the 5,297 genes predicted, 5,108 are protein-coding genes, 21 are rRNA genes, and 84 are tRNA genes. Most protein-coding genes (85.6%) were assigned a putative function. Previous characterization of the LuxI-LuxR (SplI-SplR) QS system putatively indicated the presence of a second *N*-acyl-l-homoserine lactone synthase (LuxI). The genome sequence indeed confirmed the existence of an additional pair of *luxR* and *luxI* homologues that share 45.6% and 42.3% amino acid identities with SplR and SplI, respectively. This extends the QS regulon of strain RVH1 and adds more complexity to the interplay of *N*-acyl-l-homoserine lactones (AHLs) and their production, regulation, and controlled phenotypes.

### Nucleotide sequence accession numbers.

This project has been deposited at DDBJ/EMBL/GenBank under the accession no. ARWD00000000. The version described here is ARWD01000000.
